# Electrochemical (Bio-) Sensors in Biological Applications—2nd Edition

**DOI:** 10.3390/bios15110746

**Published:** 2025-11-05

**Authors:** Sergey Shleev, Cecilia Cristea, Nina Dimcheva

**Affiliations:** 1 Department of Biomedical Science, Malmö University, SE-20506 Malmö, Sweden; 2 Biofilms Research Center for Biointerfaces, Malmö University, SE-20506 Malmö, Sweden; 3 Citizen Health Research Center, Malmö University, SE-20506 Malmö, Sweden; 4 Department of Analytical Chemistry and Instrumental Analysis, Iuliu Hațieganu University of Medicine and Pharmacy, 4 Pasteur Street, 400349 Cluj-Napoca, Romania; ccristea@umfcluj.ro; 5 Department of Physical Chemistry, University of Plovdiv “Paisii Hilendarski”, BG-4000 Plovdiv, Bulgaria; ninadd@uni-plovdiv.bg; 6 Centre of Technologies, University of Plovdiv “Paisii Hilendarski”, BG-4000 Plovdiv, Bulgaria

## 1. Introduction

The International Union of Pure and Applied Chemistry (IUPAC; https://iupac.org) defines a chemical sensor as “a device that converts chemical data, ranging from the concentration of a single sample component to complete composition analysis, into an analytically usable signal”. Chemical sensors may be classified according to the operating principle of the transducer as (i) optical devices, (ii) electrical devices, (iii) mass-sensitive devices, (iv) magnetic devices, (v) thermometric devices, (vi) electrochemical devices, and (vii) devices based on other physical properties [1]. Electrochemical sensors and biosensors (electrochemical (bio-)sensors) are analytical devices that detect target chemical species by converting a recognition event, such as a specific binding, a redox reaction, etc., into an electrical signal [2]. They typically comprise a selective receptor (often a chemical or biological recognition element) in contact with an electrode transducer, where the interaction with the analyte (e.g., oxidation or reduction at the electrode surface) produces a measurable current, voltage, or impedance change proportional to the concentration of the analyte. Electrochemical sensors that incorporate a biologically derived selective receptor, such as proteins and enzymes, antibodies, or DNA/RNA probes, are referred to as electrochemical biosensors—a class of bioelectronic devices [3].

Owing to their high sensitivity, rapid response, and low-cost instrumentation, electrochemical sensors and biosensors are among the most widely used sensor technologies [2]. In biomedical applications, these devices enable the selective and real-time detection of various biological analytes—from metabolites (e.g., glucose or lactate) to pathogens (such as bacteria or viruses) and specific ions—providing quantitative readouts for diagnostics, health monitoring, and environmental analysis.

The commercialization of electrochemical (bio-)sensors for biological applications is expanding rapidly, especially in areas such as point-of-care diagnostics [4] and wearable health monitoring [5,6]. Their affordability, sensitivity, and portability have driven market adoption [7]. However, scalability, standardization, and regulatory challenges remain key hurdles to broader implementation [8].

The Special Issue “Electrochemical (Bio-)Sensors in Biological Applications—2nd Edition” brings together nine diverse contributions that showcase both cutting-edge research and comprehensive reviews in the field of (bio-)sensor technology. The Special Issue covers advancements in both electrochemical sensors and electrochemical biosensors, highlighting their applications in various biological contexts.

## 2. Overview of Contributions

This Special Issue brings together nine diverse contributions that can be grouped into four thematic categories: Infectious Disease Detection, Clinical Diagnostics, Environmental and Industrial Monitoring, and Emerging Materials and Platforms. Each contribution is highlighted below within its respective theme, emphasizing the key targets, methodological innovations, and technological advances presented.

### 2.1. Infectious Disease Detection

Rapid and accurate detection of pathogens such as SARS-CoV-2 remains crucial for public health. Two papers in this issue advance electrochemical biosensors for infectious disease diagnostics, focusing on COVID-19. Garcia-Junior et al. developed a saliva-based COVID-19 sensor by integrating an AI-designed peptide receptor with a portable electrochemical device and machine learning analysis. Using an artificial peptide (BIAI1) optimized via the SAGAPEP platform to bind the viral spike protein, the team achieved 100% sensitivity and 80% specificity for detecting ~1.8 × 10^4^ focus-forming units of SARS-CoV-2 in saliva, with an overall accuracy of ca. 90%. A support vector machine and neural network were applied to the impedance/voltammetric data, demonstrating how artificial receptors coupled with AI can yield a non-invasive, rapid screening tool for COVID-19. Similarly, Wang et al. reported a laser-scribed graphene electrochemical sensor for SARS-CoV-2 that harnesses a novel EsterLigase-mediated nanobody immobilization to ensure proper orientation of the bioprobe. This disposable sensor strip targets the spike receptor-binding domain and transduces binding events via changes in charge-transfer resistance. The device achieved a linear detection range from 150 pM to 15 nM of viral antigen with an impressive limit of detection of 7.7 pM. By combining a facile graphene electrode platform with oriented antibody mimetics, the authors provide a promising point-of-care approach that bridges the sensitivity gap between lab-based PCR and rapid antigen tests.

### 2.2. Clinical Diagnostics

Electrochemical biosensors are powerful tools for clinical diagnostics, enabling the detection of disease biomarkers ranging from cancer-associated molecules to metabolic analytes. Three contributions in this Special Issue focus on sensor developments for healthcare applications. Chen et al. introduced an electrochemical immunosensor for detecting minimal residual disease in pediatric neuroblastoma. The sensor targets GD2, a ganglioside antigen on neuroblastoma cells, using a graphene/Au nanoparticle-coated indium tin oxide electrode functionalized with anti-GD2 antibodies. In trials with bone marrow samples, the device detected GD2-positive tumor cells down to 10^2^ cells/mL (via differential pulse voltammetry) and showed 100% agreement with clinical immunocytochemistry results in patients. This graphene/AuNP-based platform offers a rapid, quantitative tool to aid in diagnosing high-risk neuroblastoma and monitoring residual disease. Figueiredo et al. developed an amperometric enzyme biosensor to monitor galactose levels in human plasma, addressing the needs of patients with metabolic disorders, e.g., galactosemia. Their approach immobilizes galactose oxidase in a redox polymer matrix (polyvinylimidazole–polysulfostyrene) on the electrode, topped with a biocompatible poly(MPC) capping layer. This dual-polymer design improves sensor performance by securing the enzyme, facilitating electron shuttle, and blocking interferents, such as ascorbic and uric acid that are abundant in blood. The result is a more selective and antifouling galactose sensor capable of reliable measurements in complex physiological samples, paving the way for inexpensive point-of-care galactose testing. Further expanding clinical sensing, Thamilselvan et al. report a nanomaterial-based electrochemical sensor for neurological biomarkers. They synthesized cerium-doped MoS_2_ nanoflowers (Ce–MoS_2_ NFs) to simultaneously detect the neurotransmitters dopamine and epinephrine. The Ce doping yields uniquely structured MoS_2_ with a high surface area (220 m^2^/g) and intrinsic peroxidase-like catalytic activity. Deposited on screen-printed electrodes, these nanoflowers enabled the sensitive co-detection of dopamine and epinephrine, with linear ranges of 0.05–100 μM and low detection limits. The sensor’s high conductivity and catalytic properties result in simultaneous, selective measurement of multiple neurochemicals, showcasing the potential of engineered nanostructures in clinical diagnostics for neurological health.

### 2.3. Environmental and Industrial Monitoring

Beyond clinical settings, electrochemical sensors play vital roles in environmental surveillance and industrial process control. Two studies in this issue exemplify such applications by targeting environmental pollutants and bioprocess conditions. Nagpal et al. present a multifunctional sensor based on tetrapodal ZnO (t-ZnO) networks for detecting ultraviolet light and volatile organic compounds. Thanks to the unique 3D tetrapod morphology, the ZnO device achieved an extremely high UV photoresponse (9200 at 394 nm) under ambient conditions. It also operated as a gas sensor at elevated temperatures, e.g., at 350 °C, the t-ZnO network showed a clear response to 100 ppm n-butanol vapor with good repeatability and fast recovery. This “three-in-one” sensor demonstrates how a single nanostructured material can serve dual sensing modes (optical and chemical), pointing to new possibilities in environmental monitoring (e.g., ultraviolet radiation exposure and air quality) using bioengineered nanomaterials. On the industrial front, Yang et al. developed a floating capsule electrochemical system for in situ bioreactor monitoring. Their capsule-shaped device contains multiple solid-contact ion-selective electrodes and is designed to free-float in cell culture bioreactors, measuring key ions like K^+^, NH_4_^+^, Na^+^, Ca^2+^, and Mg^2+^ in real time. By wirelessly transmitting data and including on-board self-calibration, the capsule can roam the reactor, reducing biofouling and minimizing disturbances to the culture. The authors demonstrated reliable multichannel ion readings in simulated cell culture media, highlighting the system’s ability to track nutrient and metabolite levels continuously. This innovative platform exemplifies how movable, wireless sensors can improve industrial bioprocess monitoring by ensuring optimal conditions for cell growth while lowering contamination risk.

### 2.4. Emerging Materials and Platforms

Several contributions showcase emerging materials and platform technologies that push the boundaries of biosensor performance. In a comprehensive review article, Ayankojo et al. surveyed recent advances in electrochemically synthesized molecularly imprinted polymer (MIP) sensors for healthcare diagnostics. The review highlights that electropolymerization techniques allow MIPs to be polymerized directly on sensor transducers, enabling fine control of polymer film properties and robust integration of synthetic recognition elements into electrochemical devices. These MIP-based sensors have shown outstanding selectivity and stability as artificial antibodies for various disease biomarkers, and the authors discuss major trends toward translating such MIP sensors into commercial diagnostic tools. This perspective underscores the role of bioinspired polymer receptors in developing low-cost, selective diagnostics for early disease detection.

Complementing the materials focus, another editor’s choice review by Tibaduiza et al. provides a general overview of electronic “tongues” and “noses”—sensor arrays that mimic human taste and smell. This tutorial-style review covers the principles of multi-sensor platforms combined with advanced pattern recognition algorithms for identifying complex chemical mixtures. The authors illustrate how electronic tongue and nose devices have rapidly evolved and found diverse applications in environmental monitoring, food and beverage quality control, medical diagnostics, and even automotive safety. By surveying recent developments in sensing elements and data analysis methodologies, the review highlights the immense potential of these bio-mimetic sensor networks to replace or augment traditional analytical techniques, offering faster and often real-time detection across industries. Together, these two contributions emphasize how innovative materials (like MIPs and nanomaterials) and platform approaches (sensor arrays with AI) are driving the next generation of biosensors for both biomedical and environmental applications.

## 3. Conclusions and Outlooks

Recent advances in electrochemical (bio-)sensors have maintained a strong momentum, effectively linking fundamental breakthroughs with application-driven innovations in biological sensing. These devices are now being applied across a broad range of biological targets—from infectious disease pathogens to clinically relevant biomarkers—propelled by innovations in nanomaterials and microfabrication that significantly boost sensitivity and reliability. Researchers have developed novel biorecognition interfaces and improved signal transducers, leading to marked enhancements in analytical performance for detecting biological analytes. Such progress underscores the vibrancy of the field, as evidenced by a continuously growing number of publications each year and an expanding array of use cases ranging from point-of-care clinical diagnostics and robust food safety testing to real-time bioprocess monitoring.

A clear trend in the field is the transition from single-analyte laboratory sensors toward multifunctional, miniaturized, and interconnected (bio-)sensing systems. Current efforts emphasize enhanced miniaturization, wireless integration, and Internet-of-Things connectivity, alongside improved materials and (bio-)recognition elements. These advances are geared toward meeting real-world constraints such as low sample volume requirements, continuous in situ monitoring, and user-friendly operation at the point of care. Notably, this convergence of high-performance (bio-)sensors with portable and networked platforms aligns with the goals of personalized medicine. Real-time, minimally invasive (bio-)sensors—often coupled with AI-driven analytics—are increasingly capable of early disease detection and tailored health monitoring, moving healthcare from reactive testing to proactive management. The result is an emerging generation of (bio-)sensors that can simultaneously measure multiple biomarkers and transmit clinically relevant data in real time, a development poised to improve disease management and prevention greatly.

Despite this progress, several challenges must be addressed to fully realize the potential of electrochemical (bio-)sensors. A persistent gap remains between prototype demonstrations and widespread deployment. Key issues include ensuring the long-term stability of sensors in complex biofluids, reproducibility of fabrication at industrial scale, and in vivo biocompatibility for implantable or semi-implantable (wearable) devices. Biosensors rely on biologic recognition elements (enzymes, antibodies, etc.) that can degrade over time, complicating mass production and shelf-life of biodevices. Moreover, integrating (bio-)sensor output into clinical decision-making systems and digital health infrastructure will require robust data interoperability and security standards, as well as rigorous clinical validation. Addressing these technical and regulatory hurdles is critical for translating laboratory innovations into reliable products. Looking ahead, the ongoing convergence of electrochemical (bio-)sensing with advances in artificial intelligence, flexible electronics, and Internet-of-Things connectivity is expected to transform how biological information is gathered, stored and used. This interdisciplinary fusion could democratize healthcare by enabling continuous remote monitoring and personalized interventions, ultimately moving electrochemical (bio-)sensors from the laboratory bench to a cornerstone of next-generation diagnostics and health monitoring. We anticipate that the insights and innovations presented in this Special Issue will inspire further interdisciplinary collaborations aimed at transforming electrochemical (bio-)sensing from a laboratory technique into a centerpiece of next-generation diagnostics and health monitoring.
